# Can the ADC Value Be Used as an Imaging “Biopsy” in Endometrial Cancer?

**DOI:** 10.3390/diagnostics14030325

**Published:** 2024-02-02

**Authors:** Octavia Petrila, Ionut Nistor, Narcis Sandy Romedea, Dragos Negru, Viorel Scripcariu

**Affiliations:** 1 Faculty of General Medicine, The University of Medicine and Pharmacy “Grigore T. Popa”, 700115 Iasi, Romaniaviorel.scripcariu@umfiasi.ro (V.S.); 2 Department of Radiology, “Dr. C.I. Parhon” Clinical Hospital, 700503 Iasi, Romania; 3 Department of Nephrology, “Dr. C.I. Parhon” Clinical Hospital, 700503 Iasi, Romania; 4 Department of Surgery, “Dr. Iacob Czihac” Clinical Emergency Hospital, 700506 Iasi, Romania; nromedea@yahoo.com; 5 Arcadia Medical Center, 700452 Iasi, Romania; dragos.negru@umf.iasi.ro; 6 Department of Surgery, Regional Oncology Institute, 700483 Iasi, Romania

**Keywords:** MRI, endometrial cancer, ADC value, grading, myometrial invasion, lymphovascular space invasion

## Abstract

Background: The tumor histological grade is closely related to the prognosis of patients with endometrial cancer (EC). Multiparametric MRI, including diffusion-weighted imaging (DWI), provides information about the cellular density that may be useful to differentiate between benign and malignant uterine lesions. However, correlations between apparent diffusion coefficient (ADC) values and histopathological grading in endometrial cancer remain controversial. Material and methods: We retrospectively evaluated 92 patients with endometrial cancers, including both endometrioid adenocarcinomas (64) and non-endometrioid adenocarcinomas (28). All patients underwent DWI procedures, and mean ADC values were calculated in a region of interest. These values were then correlated with the tumor grading offered by the histopathological examination, which was considered the gold standard. In this way, the patients were divided into three groups (G1, G2, and G3). The ADC values were then compared to the results offered by the biopsy to see if the DWI sequence and ADC map could replace this procedure. We also compared the mean ADC values to the myometrial invasion (</>50%) and lymphovascular space invasion. Results: We have divided the ADC values into three categories corresponding to three grades: >0.850 × 10^−3^ mm^2^/s (ADC1), 0.730–0.849 × 10^−3^ mm^2^/s (ADC2) and <0.730 × 10^−3^ mm^2^/s (ADC3). The diagnostic accuracy of the ADC value was 85.71% for ADC1, 75.76% for ADC2, and 91.66% for ADC3. In 77 cases out of 92, the category in which they were placed using the ADC value corresponded to the result offered by the histopathological exam with an accuracy of 83.69%. For only 56.52% of patients, the biopsy result included the grading system. For each grading category, the mean ADC value showed better results than the biopsy; for G1 patients, the mean ADC value had an accuracy of 85.71% compared to 66.66% in the biopsy, G2 had 75.76% compared to 68.42%, and G3 had 91.66 compared to 75%. For both deep myometrial invasion and lymphovascular space invasion, there is a close, inversely proportional correlation with the mean ADC value. Conclusions: Mean endometrial tumor ADC on MR-DWI is inversely related to the histological grade, deep myometrial invasion and lymphovascular space invasion. Using this method, the patients could be better divided into risk categories for personalized treatment.

## 1. Introduction

Endometrial cancer is the second most common gynecological cancer globally and its incidence is rising in high-income countries [1]. The histological grade of the tumor is strongly associated with the risk of lymph node metastases and overall survival [2]. Histological grade is also involved in risk stratification, being an important criterion for the choice of surgical management, especially lymph node dissection [3,4]. Magnetic resonance imaging (MRI) is increasingly being used for tumor staging, like tumor size, depth of myometrial invasion, cervical stroma, and pelvic anatomical structure involvement [1]. MRI capitalizes on its high-tissue contrast resolution and reproducibility. Multiparametric MRI, particularly diffusion-weighted imaging (DWI), emerges as a valuable technique for differentiating between benign and malignant endometrial tumors [5]. DWI shows the diffusion of water molecules in tissue, which is restricted in tissue with high cellularity such as in tumors. The free motion of water in DWI is expressed by the apparent diffusion coefficient (ADC), and it has been described as helpful in differentiating between benign and malignant endometrial tumors; normal endometrium and benign endometrial lesions demonstrate significantly higher mean ADC than endometrial cancer [6]. The diagnostic accuracy of DWI with the quantitative analysis of ADC remains a subject of debate, particularly concerning the depth of myometrial invasion and tumor grade. Several studies have shown a lower apparent diffusion coefficient (ADC) value in endometrial cancer [1,7,8,9,10]. If the endometrial thickness is greater than 5 mm, biopsy is recommended and plays an important role in determining if a tumor is malignant, and if so, the histologic subtype and grade of malignancy are determined [11]. However, in postmenopausal patients, endometrial biopsy or dilatation and curettage may be challenging due to factors such as endometrial atrophy and endometrial adhesion of the need for general anesthesia [12]. In addition, several studies have shown that approximately 25% of cases have a discrepancy between the histologic grade assigned in the preoperative biopsy and the grade in the surgical specimen, indicating a tendency for underestimation [10,13,14,15,16]. This can be attributed to two factors: first, the usually small amount of tumor tissue in a biopsy may impede the assessment of tissue architecture [13,14,15,16]. Secondly, neoplasms often show a non-homogeneous distribution of cellular atypia and structural abnormalities [13,14,15,16]. Our study aimed to evaluate the possibility of predicting histologic grade, depth of myometrial invasion, and lymphovascular space invasion of endometrial cancer based on MRI parameters.

## 2. Material and Methods

### 2.1. Patient Selection

The retrospective study included 92 consecutively enrolled patients with histologically proven endometrial cancer, and the was conducted at a single institution between June 2017 and December 2021. All included patients had diagnostic MRIs before surgery, including a DWI sequence. In addition, all patients had the endometrial lesion biopsied before surgery. The exclusion criteria were as follows: no total hysterectomy, preoperative treatment with chemoradiotherapy, previous uterine surgery (subtotal hysterectomy), tumors that were too small to be visible on the MRI, MRI with obvious motion artifacts, and MRI that did not include a DWI sequence. A pathologist unaware of patients’ clinical and surgical data randomly reviewed surgical specimens on conventional HE-staining; on the final report, the following were assessed: histological cancer type (endometrioid, non-endometrioid, or mixed), grade (G1, G2, and G3), depth of myometrial invasion (<50%/>50%), cervical stromal invasion (present/absent), and lymphovascular space invasion (present/absent).

### 2.2. MR Protocol

All MRI scans were performed using a 1.5T system with an eight-channel phased array coil, with the patient in a supine position with the arms along the body. The patient was asked to fast for 6 h prior to examination and to void 1 h prior to examination. Butylscopolamine bromide was administered prior to the examination. Standard pelvic MRI was acquired with the following sequences: sagittal and axial T2w imaging, fat suppression axial T2-weighted, axial T1-weighted imaging, fat suppression axial T1-weighted imaging, axial and coronal oblique T2-weighted imaging, axial echo planar DWI (with b-values of 50, 400, and 800 s/mm^2^), and ADC mapping, which was automatically reconstructed on a post-processing workstation, dynamic contrast-enhanced T1 fat saturation, and sagittal postcontrast T1-weighted scans.

### 2.3. Image Analysis

Each study was independently evaluated by a radiologist who was blinded to the postoperative histopathological findings of endometrial cancer but was aware of a positive endometrial cancer diagnostic on biopsy.

Endometrial cancer was diagnosed when the tumor showed high signal intensity on DWI images with a b-value of 800 s/mm^2^ and low signal intensity on ADC maps. If an endometrial lesion showed hyperintensity on DWI that corresponds to a high ADC value, it was not considered a tumor. DWI inherently comprises T2-weighted scans. Therefore, the T2 shine-through effect can lead to false positive findings of restricted diffusion when isolated DW images without correlation to ADC maps are used. For each patient, a region of interest (ROI) was manually selected in the ADC map on the slice containing the largest tumor cross-section. The ROI was as large as possible to encompass the tumor and avoid contamination with normal tissue. Necrotic or hemorrhagic areas were excluded from the ROIs based on T1W imaging, T2W imaging, and fat suppression T2w imaging.

We divided the ADC values into three categories: >0.850 × 10^−3^ mm^2^/s ADC 1, which corresponds to low-cellularity tumors; between 0.730 and 0.849 × 10^−3^ mm^2^/s ADC 2, which corresponds to intermediate cellularity tumors; and <0.730 × 10^−3^ mm^2^/s ADC 3, which corresponds to high cellularity tumors.

The depth of myometrial invasion was classified as the International Federation of Gynecology and Obstetrics (FIGO) stage 1A (endometrial cancer is limited in the endometrium or invades up to 50% of the myometrium) and FIGO 1B (endometrial cancer invades more than 50% of the myometrium).

### 2.4. Statistical Analysis

Statistical analysis was performed with SPSS version 26 for Windows using descriptive analysis, frequency analysis and cross-tabulation, chi-square test, and Pearson correlation.

## 3. Results

### 3.1. Patient Characteristics

The mean age of patients was 63 years old (63 ± 20 years old). As summarized in TABEL 1, the most common symptom for referral to the hospital was abnormal vaginal bleeding (98.9%). Upon pre-operative biopsy, the pathologist classified 73 cases as endometrioid, 10 as non-endometrioid, and 7 were mixed types of endometrial cancer. In two cases, there was atypic hyperplasia with intraepithelial neoplasia.

All cases were confirmed via histopathology after surgery, consisting of total hysterectomy with bilateral salpingo-oophorectomy; lymphadenectomy was performed in 80 cases. Most cases (64/92) were endometrioid adenocarcinomas, 15 of which were adenocarcinomas with squamous differentiation and 2 of which were adenocarcinomas with mucinous differentiation. Different histological types were observed in the remaining 28 cases (Table 1). The histological grades of endometrial cancers are 37 G1, 31 G2, and 24 G3.

Of the 92 patients included in our study, during the 2-year follow-up, 9 presented with metastases or tumor recurrence. In six cases, the tumors were poorly differentiated, one case was moderately differentiated, and two were well differentiated.

### 3.2. MRI Findings

**Figure 1:**
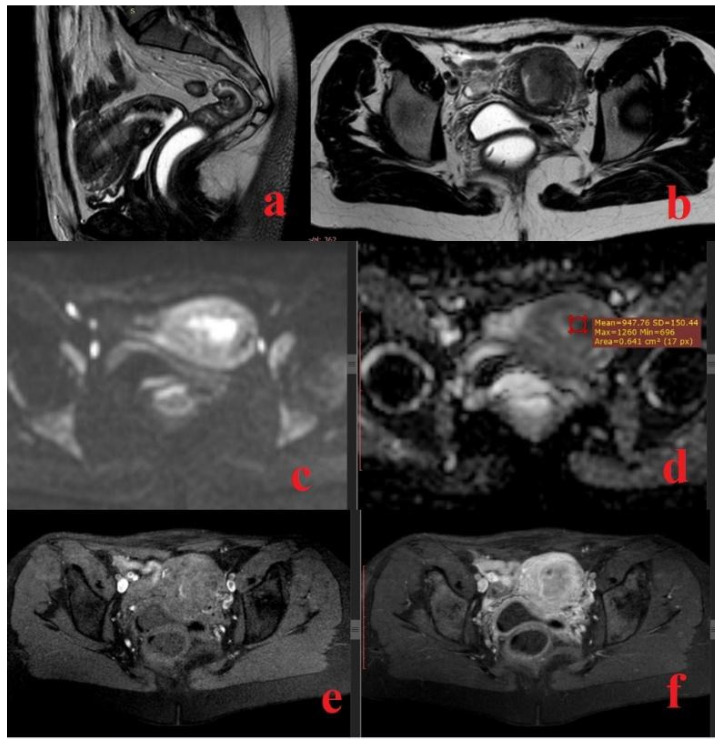
A 46-year-old woman with histopathologically proven low-grade endometrioid endometrial cancer; sagittal T2-weighted fast spin-echo image (**a**) and axial view (**b**) show a hypointense mass in the endometrial cavity, infiltrating more than 50% of the myometrial thickness; axial DWI (**c**) and ADC map (**d**) show a hyperintense mass on high b value image (b = 800 s/mm^2^) corresponding to a low-intensity mass on the derived ADC map; the ADC value is 0.948 × 10^−3^ mm^2^/s; axial fat-saturated spin-echo T1-weighted image (**e**) and gadolinium-enhanced fat-saturated spin-echo T1-weighted image (**f**) show the tumor as a slightly enhanced mass.

**Figure 2:**
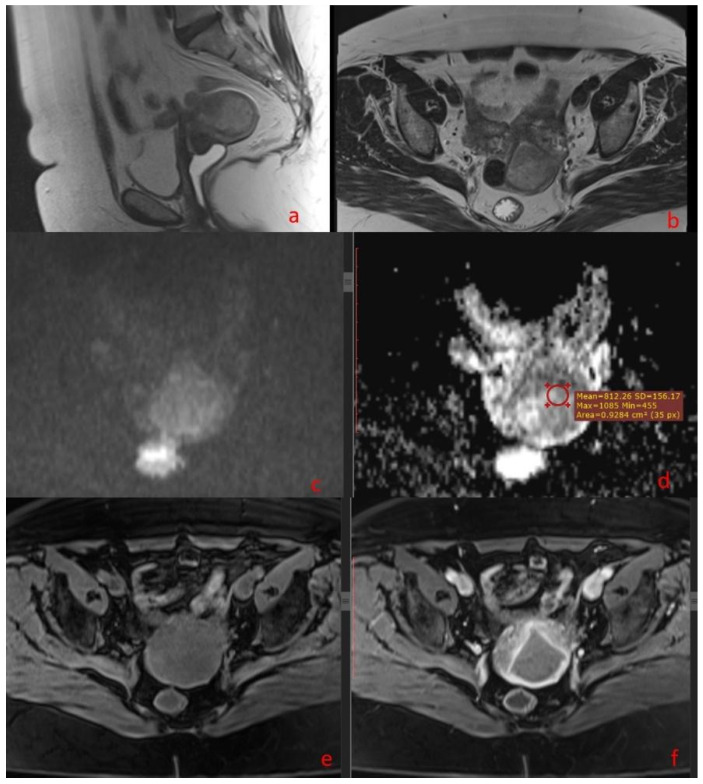
A 66-year-old woman with histopathologically proven medium-grade endometrioid endometrial cancer; sagittal T2-weighted fast spin-echo image (**a**) and axial view (**b**) show a hypointense mass in the endometrial cavity, infiltrating more than 50% of the myometrial thickness and the cervical stroma; also, on the axial view, (**b**) there is an intramural leiomyoma with no mass effect on the endometrial cavity; axial DWI (**c**) and ADC map (**d**) show an inhomogeneous hyperintense mass on high b value image (b = 800 s/mm^2^) corresponding to a low-intensity mass on the derived ADC map; the ADC value is 0.812 × 10^−3^ mm^2^/s; axial fat-saturated spin-echo T1-weighted image (**e**) and gadolinium-enhanced fat-saturated spin-echo T1-weighted image (**f**) show the tumor as a slightly enhanced mass.

**Figure 3:**
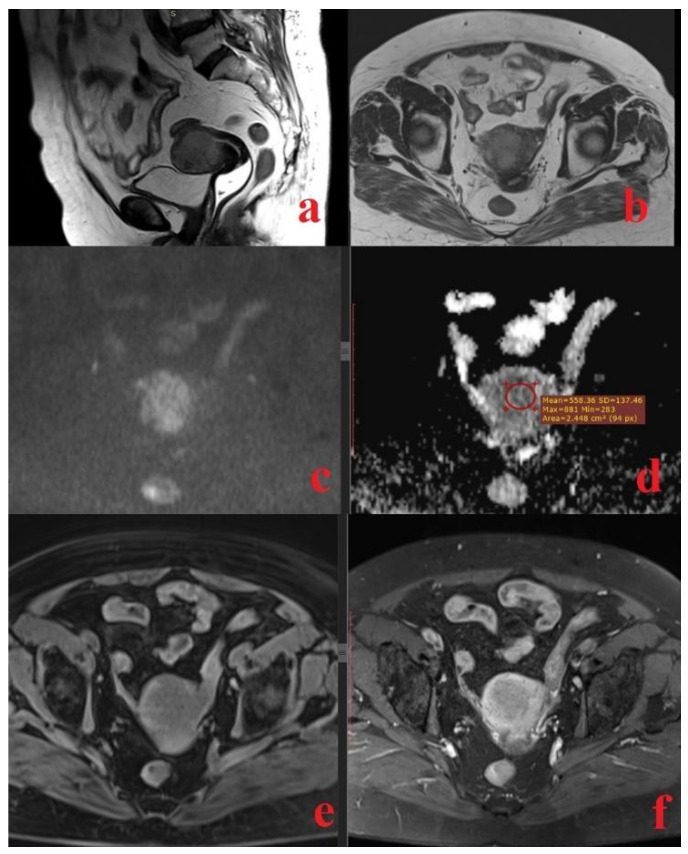
A 75-year-old woman with a histopathologically proven high-grade endometrial cancer (mixed type endometrioid and serous); sagittal T2-weighted fast spin-echo image (**a**) and axial view (**b**) show a hypointense mass in the endometrial cavity, infiltrating more than 50% of the myometrial thickness and cervical stroma and invading the uterine serosa; the tumor; axial DWI (**c**) and ADC map (**d**) show a hyperintense mass on high b value image (b = 800 s/mm^2^) corresponding to a low-intensity mass on the derived ADC map; the ADC value is 0.558 × 10^−3^ mm^2^/s; axial fat-saturated spin-echo T1-weighted image (**e**) and gadolinium-enhanced fat-saturated spin-echo T1-weighted image (**f**) show the tumor as a slightly enhanced, inhomogeneous mass.

The mean ADC value calculated for the well-differentiated endometrial cancer (G1) based on post-surgery histology was 0.926 × 10^−3^ mm^2^/s, 0.796 × 10^−3^ mm^2^/s for moderately differentiated (G2), and 0.670 × 10^−3^ mm^2^/s for poorly differentiated (G3), with a total average of 0.797 × 10^−3^ mm^2^/s (Figure 1, Figure 2 and Figure 3).

We divided the ADC values into three categories using the mean ADC for each tumor grade and removing the standard deviation: >0.850 × 10^−3^ mm^2^/s (ADC1), between 0.730 and 0.849 × 10^−3^ mm^2^/s (ADC2), and <0.730 × 10^−3^ mm^2^/s (ADC3). Out of the 35 cases in which the ADC value was labeled as ADC1, in the histopathological exam, only 5 cases were false positive (1 being grade 3 and 4 being grade 2), so the accuracy is 85.71% for ADC1. Out of the 33 cases labeled as ADC2, 7 cases were presented in histopathological exam G1 and 1 presented in G3, with an accuracy of 75.76%. In total, 24 cases were labeled as ADC3, with a histopathological exam accuracy of 91.66%, and only 2 cases were in histopathological exam G2. A total of 77 patients were correctly evaluated regarding the grading system using the manually traced mean ADC value. 

When talking about presurgical biopsy in only 52 cases out of 92 patients enrolled (56.52%), it offered tumoral grade evaluations. Also, for each grading category, the concordance between preoperative and postoperative assessments is lower than the ADC grading system. Thus, for G1, the biopsy concordance is 66.66% compared to 85.71%; for G2, it is 68.41% compared to 75.76%, and for G3, it is 75% compared to 91.66%. Overall, there was a diagnostic concordance of 69.23% for the 52 cases in which the biopsy result offered a grading evaluation, but if we take into consideration all the cases from the study, the correct preoperative grading evaluation was only 39.13%.

Regarding the depth of myometrial invasion, there is a high correlation that is inversely proportional between the mean ADC value and the depth of myometrial invasion (*p* = 0.001) without a clear cut-off value (Table 2).

Another important prognostic factor evaluated in our study was the correlation between the mean ADC value and lymphovascular space invasion. There is a strong correlation that is inversely proportional between the ADC value and lymphovascular invasion, with a significance level of 0.01 (*p* < 0.0001) and without a clear cut-off value (Table 3).

As we can see in Table 4, there is a strong inversely proportional correlation between tumor grade and the depth of myometrial invasion and lymphovascular space invasion. There are also few cases in which patients that only have superficial myometrial invasion had lymphovascular space invasion as a risk factor for lymph node involvement.

## 4. Discussion

We studied the diagnostic roles of mean tumor ADC from MR-DWI in patients with endometrial cancer and showed that the mean ADC value is inversely correlated with the tumoral grade, depth of myometrial invasion, and lymphovascular space invasion. However, we did not find a clear cut-off ADC value.

DWI can be influenced not only by tumor cell density but also by factors such as tumor proliferation, perfusion, extracellular space relative to normal tissue, and stromal condition. The correlation between mean ADC and tumor cellularity varies across different organs, often leading to discrepancies [5,17,18,19].

In cases where endometrial biopsy or curettage is challenging, MR-DWI can be a valuable diagnostic tool. MR-DWI is a sensitive method for assessing the depth of myometrial invasion, eliminating the need for invasive diagnostic procedures and minimizing the risk of unnecessary overtreatment [12,13,14,15,16]. The role of ADC in characterizing endometrial tumors remains a subject of debate without consensus yet. In a study of 18 patients, Tamai et al. found that normal endometrium has a higher ADC value than endometrial cancer. Additionally, they found a progressive decrease in ADC values with an increase in tumor grade. Endometrial carcinoma typically exhibits diffusion restriction, appearing as hyperintense compared to the surrounding myometrium and exhibiting hypointense signal properties in the ADC mapping. However, other studies found no correlation between tumor ADC values and histological tumor grade [6,9,20,21,22]. The mean ADC value for endometrial cancer was 0.797 × 10^−3^ mm^2^/s; the one found in the present study is comparable to that found by Fujii et al., Shen et al., and Takeuchi et al., and it is much lower than the reported values for normal endometrium reported by Çavusoğlu et al. [1,5,21,23,24]. In our G3 group, we also included carcinosarcomas (four patients) with a mean of 0.624 × 10^−3^ mm^2^/s, which was lower than the mean for the total G3 groups (0.670 × 10^−3^ mm^2^/s), unlike other studies from the literature that reported carcinosarcomas with higher ADC values due to a higher frequency of necrosis and epithelial cystic components. Low-grade endometrial carcinomas can sometimes exhibit “normal-appearing” glands resembling a morphologically normal endometrium and may be associated with or preceded by endometrial hyperplasia. These low-grade endometrial carcinomas typically display low cellularity and increased water molecule motion, leading to higher ADC values. Conversely, high-grade endometrial carcinomas, characterized by high cellular density, are expected to have lower ADC values [7,24].

The association between high-risk tumors and low ADC values has been explained by factors such as increased tumor cellularity, proliferation, perfusion, and reduced extracellular space. Previous studies have reported varying test characteristics for tumor grading by MR-DWI, ranging from a sensitivity between 55% and 100% and a specificity between 70% and 89% [5,17,18,19]. A meta-analysis by Yan et al. demonstrated moderate diagnostic performance with a pooled sensitivity of 77% and a specificity of 73%, respectively. However, there is ongoing debate regarding the use of ADC values to differentiate tumor grades, as there is a considerable overlap observed between low-grade and high-grade tumors. This can be attributed to other factors involved in determining tumor grades, such as nuclear atypia and pathological proteins, which cannot be evaluated solely using the ADC map and calculated values [3].

The problem with ADC value measurements is that it depends on drawing the ROI on the ADC map, which has a low signal-to-noise ratio. Also, it is difficult to draw an accurate ROI along with an unclear tumor boundary because some patients have small tumors that were developed in the atrophic uterus and can also be affected by the position of the uterus [3].

The prognostic factors for endometrial cancer are the age of the patient, stage, tumor grade, histology, depth of myometrial invasion, cervical involvement, and lymph node metastasis. The detection of preoperative myometrial invasion is very important in treatment planning (the width of lymph node dissection) and predicting patients’ prognosis [10]. In the latest FIGO classification, stage IA is limited to the endometrium or invades less than 50% of the myometrium, while stage IB tumors invade equal or more than 50% of the myometrium. MRI serves as a reliable tool for assessing the depth of myometrial invasion in endometrial cancer. Additionally, Bonatti et al. found that the presence of deep myometrial invasion (>50% of myometrial thickness) on MRI is significantly associated with high-grade (G2–G3) neoplasms based on subsequent histological analysis. In their study, deep myometrial invasion was observed in 67% of G3 lesions, 61% of G2 lesions, and 39% of G1 lesions. This correlation is a direct result of the less infiltrative growth pattern typically seen in low-grade tumors that tend to exhibit fewer instances of lymphovascular invasion compared to high-grade tumors. Moreover, deep myometrial invasion is significantly linked to the presence of nodal metastasis, with the risk of pelvic or paraaortic lymph node metastasis increasing 6–7× in this case [1,2].

The current standard surgical procedures and staging are total hysterectomy, bilateral salpingo-oophorectomy, and peritoneal cytology. The need for routine pelvic and paraaortic lymph node dissection remains a topic of debate for low-risk patients, while it is generally planned for high-risk patients with deep myometrial invasion, cervical extension, or extrauterine disease, as these patients have a higher risk of nodal metastasis. However, considering the high incidence of systemic diseases such as obesity, hypertension, and diabetes in postmenopausal patients, the need to avoid overtreatment in low-risk patients is important. Therefore, accurate assessments of the histologic type and degree of myometrial invasion play a crucial role in treatment planning [1,2].

Consistent with our study, Husby et al. reported a significantly lower mean ADC value in tumors with deep myometrial invasion (ADC value of 0.75 × 10^−3^ mm/s) compared to tumors with superficial myometrial invasion (ADC value 0.85 × 10^−3^ mm^2^/s) [25]. However, other studies by Rechichi et al. and Lin et al. found no statistically significant difference in mean ADC values between FIGO stage 1A and FIGO 1B lesions [6,22].

Previous studies have indicated that ADC values can be used to assess deep myometrial invasion, with a sensitivity and specificity ranging from 47.6 to 78% and 57 to 88%, respectively. In the meta-analysis conducted by Yan, moderate sensitivity and specificity for evaluating the depth of myometrial invasion were demonstrated. DWI can provide a clearer visualization of the tumor and the boundary with the normal myometrium compared to T2-weighted imaging. A high b-value DWI shows a high contrast-to-noise ratio. DWI exhibits both a sensitivity and specificity of 86% for detecting deep myometrial invasion, showing better diagnostic performance compared to ADC values [3].

Our study has some limitations. First of all, it has a small sample size and a retrospective design; further prospective studies are required to validate the present results in a larger population and for other histological subtypes. Second, only 1.5T equipment was used in this study, and better tissue contrast might be obtained using a 3T machine. Another limitation might reside in the fact that the ADC values’ distribution was calculated using single slice modality and not over the entire tumor volume, making it less reproducible and generating variations between measurements depending on the placement and dimension of the ROI; on the other hand, volumetric evaluation might have been extremely time consuming and, therefore, of poor utility in everyday clinical practice.

## 5. Conclusions

In women with endometrial cancer, there is a strong inverse correlation between mean tumor ADC from MR-DWI and histological grade, myometrial infiltration, and lymphovascular space invasion. The mean ADC presents a higher value in determining tumor grade than the biopsy, which sometimes did not offer this information, being a method that deserves further research and development, as it may become a criterion to consider when establishing therapeutic protocols.

## Figures and Tables

**Table 1:** Summary of patient characteristics and histopathology data.

Characteristics	Patients (n: 92)		
Age	63 years old (43–84 years old)		
Symptoms			
Bleeding	98.9%		
Pelvic pain	8%		
Fatigue	9%		
Weight loss	2%		
Histology	Biopsy	Post-surgical histology	
Endometrioid adenocarcinoma	73	64	
Non-endometrioid adenocarcinoma	10	28	
Mixed types	7	n/a	
Atypic hyperplasia	2	n/a	
Tumor grade	Biopsy	Post-surgical histology	ADC
G1	26	37	35
G2	14	31	33
G3	12	24	24
Missing	40		
Tumor characteristics		MRI	Post-surgical histology
Tumor maximum dimension	<2 cm	15	n/a
	>2 cm	77	n/a
Depth of myometrial invasion	<50%	50	43
	>50%	42	49
Cervical stroma involvement	Negative	66	68
	Positive	26	24
Lymph node involvement	Negative	70	66
	Positive	22	13
	Lymphadenectomy not performed		13

**Table 2:** Pearson correlation proves a high correlation that is inversely proportional between the mean ADC value and the depth of myometrial invasion.

		ADC Value	Histologically Proven Myometrial Invasion
ADC value	Pearson correlation	1	−0.360 **
	Sig. (2-tailed)		0.000
	N	92	92
Histologically proven myometrial invasion	Pearson correlation	−0.360 **	1
	Sig. (2-tailed)	0.000	
	N	92	92

** Correlation is significant at the 0.01 level (2-tailed).

**Table 3:** Pearson correlation exhibits a high correlation that is inversely proportional between the mean ADC value and lymphovascular space invasion.

		ADC Value	Lymphovascular Space Invasion
ADC value	Pearson correlation	1	−0.522 **
	Sig. (2-tailed)		0.000
	N	92	92
Lymphovascular space invasion	Pearson correlation	−0.522 **	1
	Sig. (2-tailed)	0.000	
	N	92	92

** Correlation is significant at the 0.01 level (2-tailed).

**Table 4:** Tumor grade and the depth of myometrial invasion and lymphovascular space invasion.

	Total Number	<50% Myometrial Invasion	>50% Myometrial Invasion	Lymphovascular Space Invasion	Patients with <50% Myometrial Invasion, but with Lymphovascular Space Invasion
G1	37	22	15	7	3
G2	31	10	21	17	2
G3	24	10	14	19	5

## Data Availability

The datasets generated and analyzed during the current study are available from the corresponding author upon reasonable request.
